# Preliminary experience of the robot-assisted laparoscopic excision of a retroperitoneal mass: A case report

**DOI:** 10.3892/ol.2014.2533

**Published:** 2014-09-12

**Authors:** QIN LIU, XINJING WANG, BAIYONG SHEN, LIANGCHAO ZHAO, QIAN ZHAN, SHULIN ZHAO, CHENLEI WEN, XIAXING DENG, CHENGHONG PENG, HONGWEI LI

**Affiliations:** Department of General Surgery, Ruijin Hospital, Shanghai Jiaotong University School of Medicine, Shanghai Institute of Digestive Surgery, Shanghai 200025, P.R. China

**Keywords:** retroperitoneal mass, pheochromocytoma, extra-adrenal, robot-assisted, laparoscopic

## Abstract

The aim of the present study was to report the initial clinical experience of adopting the da Vinci Surgical System (Intuitive Surgical, Inc., Sunnyvale, CA, USA) to perform a retroperitoneal tumor resection. The patient was a 56-year-old female who presented with a five-year history of hypertension. Abdominal dynamic computed tomography (CT) and positron emission tomography-CT scans revealed a mass measuring ~6 cm in diameter that was located anterior to the abdominal aorta, and between the abdominal aorta and the inferior vena cava (at the level of the third lumbar vertebra). The tumor was excised via a five-port, robot-assisted, transperitoneal laparoscopic approach. Careful dissection of the tumor away from the abdominal aorta and the inferior vena cava was accomplished without resulting in major vascular injury. There were no complications and the patient was discharged in a good condition on the eleventh postoperative day. Pathological analysis of a tumor specimen demonstrated a benign pheochromocytoma (PHEO). During the three-month follow-up, no recurrence was identified through CT scans or measurement of the patient’s endocrine hormone levels. Thus, the da Vinci robot-assisted laparoscopic system may be safely employed in the treatment of extra-adrenal PHEOs that occur in difficult locations for which a laparoscopic surgical excision may be challenging.

## Introduction

Laparoscopic minimally invasive surgical techniques are not routinely adopted for the excision of retroperitoneal tumors, which are often located adjacent to vital organs and blood vessels. Indeed, minimally invasive surgery is even more rarely adopted for retroperitoneal tumors that are located between the vena cava and the abdominal aorta (1).

A pheochromocytoma (PHEO) is a type of catecholamine (CA)-producing neuroendocrine tumor, which originates in the chromaffin cells of the paraganglia. Worldwide, the incidence of PHEO ranges between 0.005 and 0.1%. In total 10% of PHEOs occur at extra-adrenal sites. Extra-adrenal paraganglionic tumors are termed paragangliomas or extra-adrenal PHEOs (2) and are primarily derived from the organ of Zuckerkandl proximal to the aorta. Diagnosis and therapy remain complicated and there are no histological criteria for distinguishing between benign and malignant tumors. Complete surgical resection is the first choice approach. Radioactive isotope treatment with [^131^I]-MIBG, systemic chemotherapeutic intervention and genetic analysis under development (3,4). Recently, laparoscopic surgery has become the standard treatment for benign and malignant PHEO. However, despite recent advancements, laparoscopy is limited by the degree of motion that can be achieved by instruments (4,5). In the current study, a preliminary report of results from a robot-assisted minimally invasive surgical procedure on an extra-adrenal PHEO, which was located between the abdominal aorta and the inferior vena cava is presented. Written informed consent was obtained from the patient.

## Case report

### Patient information

**Figure 1:**
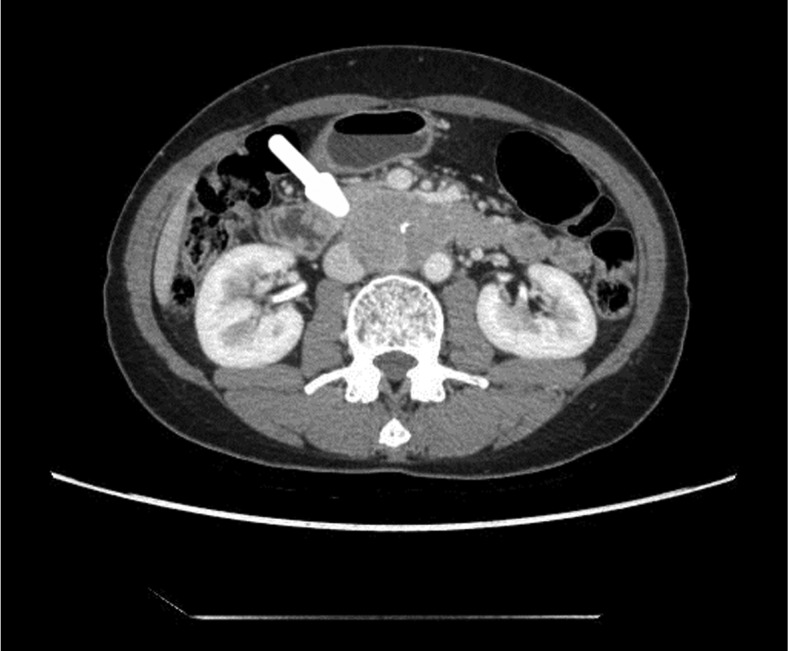
Abdominal computed tomography scan revealing a 5×6cm tumor. Arrow indicates the tumor.

The patient was a 56-year-old female with a five-year history of hypertension. The patient’s preoperative blood norepinephrine (NE) and epinephrine (E) levels were 9,866.9 pg/ml (normal range, 19.0–121 pg/ml) and 188 pg/ml (normal range, 14.0–90.0 pg/ml), respectively, which were accompanied by a headache, palpitations and sweating. The highest blood pressure reading was 240/110 mmHg (normal range, 100–120/60–80 mmHg). Preoperative positron emission tomography-computed tomography (PET-CT), and CT angiography (CTA) revealed a round-shaped mass that was ~6 cm in diameter and located anterior to the abdominal aorta, proximal to the inferior vena cava at the level of the third lumbar vertebra (termed L3). The tumor was in close proximity to, and located between, the abdominal aorta and the inferior vena cava; furthermore, the mass was posterior to the duodenum and the uncinate process of the pancreas, as well as being tightly adhered to the inferior vena cava. The tumor had a rich blood supply and no obviously swollen retroperitoneal lymph nodes or distant metastases were observed (Fig. 1).

Prior to surgery, the patient was suspected to have an ectopic PHEO and was treated using orally administered Cardura (doxazosin; Pfizer, Inc., New York, NY, USA; 4 mg, once a day for seven days) to lower and control the elevated blood pressure to 120/80 mmHg. The preoperative assessment of the patient’s heart and lung function indicated that she was able to tolerate the surgical procedure. The patient had no previous history of upper abdominal surgery, however, had previously undergone a cesarean section.

### Preoperative preparation and anesthesia

The preoperative preparations were similar to those of laparotomic resections of ectopic adrenal PHEOs (7). The patient was placed in the left lateral decubitus position (with the right side elevated ~70°) under general anesthesia.

### Installation of the da Vinci Robotic Surgical System (Intuitive Surgical, Inc., Sunnyvale, CA, USA)

**Figure 2:**
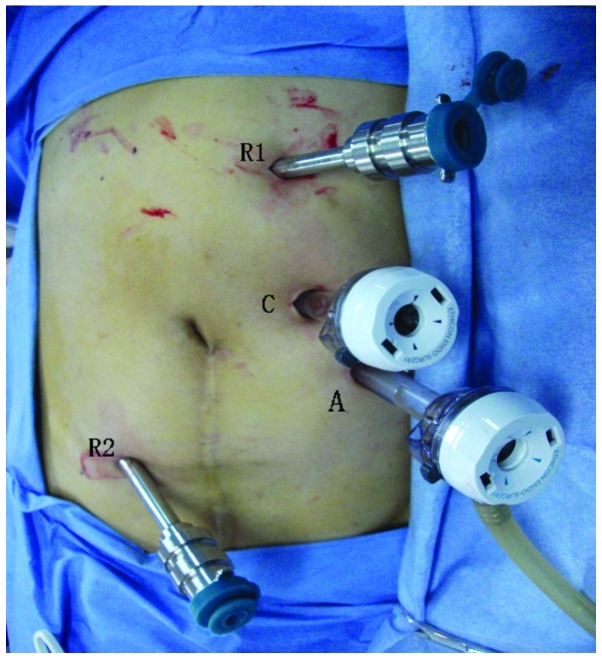
Trocar placement: R1, robotic arm no. 1; R2, robotic arm no. 2; C, camera port; A, accessory port.

Based on the preoperative positioning and CT images, the pneumoperitoneum was established by puncturing the point of intersection of the left mid-clavicular line and the costal margin. A trocar was inserted to establish the camera port, and a robotic camera was inserted to determine whether tumor metastases were present in the abdominal cavity and whether there were obvious indications against performing the surgery. A four-port approach was used to insert the remaining trocars (Fig. 2). The robotic arm tower was mounted and the operating arms were installed. Subsequently, the accessory port was positioned between robotic arm no. 1 and the camera port.

### Tumor resection

The lower abdominal adhesions were dissected, a Kocher incision was made to mobilize the duodenum, and the pancreatic head and the duodenum were shifted to the left. This was to facilitate the investigation of the association between the tumor and major blood vessels, such as the abdominal aorta and the inferior vena cava.

**Figure 3:**
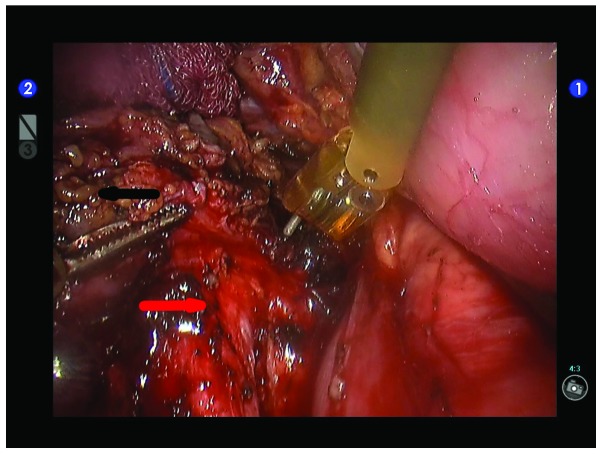
Dissection of the tumor away from the abdominal aorta. The black arrow indicates the tumor and the red arrow indicates the abdominal aorta.

The tissue that was posterior to the tumor was progressively freed from right to left and from bottom to top until the level of the vena cava was reached. The abdominal aorta was protected, using an electrical hook and bipolar coagulator to seperate the tumor from the abdominal aorta and inferior vena cava, while the tumor was dissected away from it (Fig. 3). The blood vessels that nourished the tumor were ligated using an electric hook or titanium clips, and the tumor was freed from the vena cava and the renal vein. The tumor was observed to have densely infiltrated the inferior vena cava; however, the intact tumor was completely dissected from the inferior vena cava while the major blood vessels around the tumor (such as the right gonadal vein) were carefully protected. Finally, the adhesions between the tumor and the prevertebral tissue were dissociated.

The tumor was placed in a specimen bag and a separate incision was made to remove the specimen bag. The specimen subsequently underwent pathological examination. In addition, the wound surfaces were washed, examined and subjected to strict hemostasis.

### Drainage tube placement

A drainage tube was placed in the surgical area between the abdominal aorta and the vena cava, and exited via the trocar hole.

## Results

**Figure 4:**
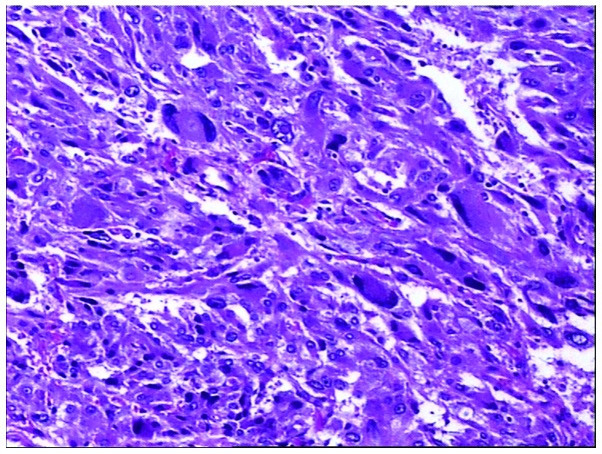
Tumor tissue slice for pathological analysis. Stain, hematoxylin and eosin; magnification, ×100.

There were no complications during surgery, the intraoperative blood pressure was stable (without significant fluctuation) and there was no requirement to convert to a laparotomy. The patient’s drainage tube was removed after seven days, and the patient was discharged 11 days following the surgery. The postoperative pathological report obtained via analysis of a tissue slice indicated a diagnosis of paraganglioma with visible extracapsular vascular invasion (Fig 4).

The patient was followed up for three months and no clear tumor recurrence was identified by CTA during the follow-up examinations. The blood NE and E levels were restored to within their normal ranges of 19.0–121 and 14.0–90.0 pg/ml, respectively.

## Discussion

The clinical manifestations of extra-adrenal PHEOs are diverse, including paroxysmal symptoms (palpitations, headache, sweating and pallor), hypertension, an adrenal or abdominal mass, high blood sugar, lactic acidosis, weight loss and other symptoms of the metabolic syndrome (8). Extra-adrenal PHEOs commonly exhibit three typical symptoms, which are headaches, palpitations and sweating. In addition, 80–100% of patients have persistent or paroxysmal hypertension (9). The clinical manifestations depend on the relative quantities of E, NE and dopamine that are secreted by the tumor. The disease symptoms may exhibit a sudden onset that generally persists for several minutes to 1 h and is precipitated by hyperplasia, constipation, abdominal pressure, or a variety of therapeutic agents (such as glucagon, contrast agents, tyramine, metoclopramide and tricyclic antidepressants) (10). Therefore, intraoperative blood pressure monitoring and the reduction of tumor stimulation present as challenges for anesthesiologists and surgeons.

The emergence of laparoscopic techniques enables the removal of adrenal tumors in a minimally invasive manner and the laparoscopic resection of adrenal PHEOs has been used in clinical practice for many years (11). An investigation conducted outside of China by Gill (12) demonstrated that laparoscopic surgery leads to milder stimulation of functional PHEOs when compared with a laparotomy; furthermore, the CA hormone levels that are secreted during surgery are lower. However, the difficulties and limitations of laparoscopic resections of adrenal PHEOs arise when blood vessels require handling during the surgical procedure, when the tumor is located within anatomical structures that are located deep in the abdominal cavity (particularly for retroperitoneal ectopic PHEOs) and when the tumor is excessively large (11).

The da Vinci robot-assisted laparoscopic surgical system is based on laparoscopic surgery and possesses various advantages, such as clear three-dimensional images, the EndoWrist^TM^ (Intuitive Surgical, Inc.) instrument (which mimics the human wrist with seven degrees of freedom), hand-tremor elimination, motion scaling and motion indexing (13). This system generates clear three-dimensional images, and achieves an accurate three-dimensional depth of field using a high resolution, thus enabling the surgeon to acquire a visual field and an operating position, which approximates open surgery. In particular, due to the narrow retroperitoneal anatomical space that restricts the area for conducting laparotomic surgery, the robot-assisted laparoscopic surgical system provides natural advantages with regard to handling the complex anatomical structures and the areas adjacent to any important blood vessels (14). Furthermore, this system overcomes certain issues associated with the original laparoscopic techniques, for example, hand tremors and a low degree of freedom when operating the instruments. Therefore, the system achieves more precise separation and dissection of tumors that are located within anatomical structures deep in the abdominal cavity. Additionally, this method facilitates the minimally invasive resection of tumors that are adjacent to major blood vessels in the abdominal cavity (such as the abdominal aorta, inferior vena cava, renal artery and the renal vein) and is considered to be safer and more stable than traditional laparoscopic surgical procedures. This novel technique fully inherits the advantages of laparoscopic surgery, reduces the stimulation of endocrine tumors, ligates and dissects the tumor-feeding blood vessels in a timely manner and reduces the incidence of intraoperative complications.

Currently there are a number of reports concerning robot-assisted laparoscopic resection for adrenal tumors, such as PHEO (15,16). As the occurrence of extra-adrenal paraganglioma is rare, reports regarding this type of tumor are also uncommon, to the best of our knowledge, only Lehrfeld *et al* (14) have presented their experience of conducting a robot-assisted excision of a retroperitoneal mass in 2010.

In the present case, due to the specific anatomical location of the tumor, which was proximal to the inferior vena cava and abdominal aorta, the traditional laparoscopic instruments were limited by the deep anatomical structures and the omental mesenteric vascular occlusion. Generally, surgeons are unable to avoid avulsing aspects of the mesenteric vessels, which may affect the blood supply to regions of the intestines, cause ischemia, delay postoperative functional recovery and potentially lead to partial bowel resection. Whereas, robot-assisted laparoscopic surgery systems are able to accurately dissect the tumor from the surrounding tissue while the feeding vessels surrounding the tumor (particularly those with an abundant blood supply) may be stanched using an ultrasonic scalpel and electrocoagulation; however, during traditional laparoscopic surgery, there is practically no ligation or suturing. When flexible robotic arms are adopted for surgery, the vessels surrounding the tumor may be smoothly manipulated to achieve satisfactory hemostasis and a successful dissection.

As a result of our past experience (17–19), the robot-assisted laparoscopic surgery system is considered to be efficacious when adopted for the excision of benign and borderline retroperitoneal tumors that have no significant adhesions with surrounding tissues, such as fibrous tumors, teratoma and particularly for tumors that are in close proximity to major vessels. The biological behavior and pathological nature of malignant tumors varies, however, if the capsule is complete, and the radiological data and intraoperative findings demonstrate no significant infiltration, minimally invasive surgery can also be considered. The robot-assisted system may facilitate the reduction of surgical trauma and the influence on the body’s immune system, thus promoting postoperative recovery, enhancing the quality of life of the patients and improving all possible outcomes.

In conclusion, retroperitoneal ectopic adrenal PHEOs are particularly rare. This type of tumor is located deep within the abdominal cavity, often in the deep retroperitoneal anatomical structures. Consequently, PHEOs are complex to treat, even when adopting the laparotomic surgical approach. The associated risk is particularly significant for tumors that affect the endocrine functions. The robot-assisted laparoscopic surgical system provides a novel, minimally invasive solution for treating retroperitoneal tumors. Furthermore, this technique reduces stimulation of the tumor and prevents or minimizes complications. To the best of our knowledge, the patient in the present study is currently the first known patient in China that has successfully undergone a robot-assisted resection of a retroperitoneal ectopic adrenal PHEO, making this study particularly significant.
